# Expression of miR-34c induces G2/M cell cycle arrest in breast cancer cells

**DOI:** 10.1186/1471-2407-14-538

**Published:** 2014-07-26

**Authors:** Chandrani Achari, Sofia Winslow, Yvonne Ceder, Christer Larsson

**Affiliations:** Department of Laboratory Medicine, Translational Cancer Research, Lund University, Medicon Village, Building 404:C3, 223 81 Lund, Sweden

**Keywords:** Breast cancer cells, miRNA-34c, CDC23, PKCα, Cell cycle arrest

## Abstract

**Background:**

MicroRNA-34 is a family of three miRNAs that have been reported to function as tumor suppressor miRNAs and show decreased expression in various cancers. Here, we examine functions of miR-34c in basal-like breast cancer cells.

**Methods:**

Data from The Cancer Genome Atlas (TCGA) were used for evaluation of expression in primary breast cancers. Cellular processes affected by miR-34c were investigated by thymidine incorporation, Annexin V-assays and cell cycle analysis using breast cancer cell lines. Effects on potential targets were analyzed with qPCR and Western blot.

**Results:**

TCGA data revealed that miR-34c was expressed at lower levels in basal-like breast cancer tumors and low expression was associated with poor prognosis. Ectopic expression of miR-34c in basal-like breast cancer cell lines resulted in suppressed proliferation and increased cell death. Additionally, miR-34c influenced the cell cycle mainly by inducing an arrest in the G2/M phase. We found that expression levels of the known cell cycle-regulating miR-34 targets *CCND1*, *CDK4* and *CDK6*, were downregulated upon miR-34c expression in breast cancer cell lines. In addition, the levels of *CDC23*, an important mediator in mitotic progression, were suppressed following miR-34c expression, and siRNAs targeting *CDC23* mimicked the effect of miR-34c on G2/M arrest. However, protein levels of *PRKCA*, a predicted miR-34c target and a known regulator of breast cancer cell proliferation were not influenced by miR-34c.

**Conclusions:**

Together, our results support the role of miR-34c as a tumor suppressor miRNA also in breast cancer.

**Electronic supplementary material:**

The online version of this article (doi:10.1186/1471-2407-14-538) contains supplementary material, which is available to authorized users.

## Background

MicroRNAs (miRNAs) are small (~22 nt) non-coding RNAs of importance for protein level regulation. They act by interacting with the 3’UTR of the target mRNA which may cause mRNA degradation or translational inhibition [1, 2]. Several miRNAs have been associated with processes involved in cancer progression, *e.g*. proliferation, differentiation, apoptosis and tumorigenesis [3] and miRNAs have been classified as both oncogenic and tumor suppressive [4].

The miR-34 family consists of three homologous miRNAs located at chromosome 1 (miR-34a) and chromosome 11 (miR-34b/c) at positions frequently deleted in solid tumors, *e.g*. neuroblastoma, breast, prostate and lung cancer [5–9]. Several reports have also pointed out a decreased expression of miR-34 in numerous malignancies, such as miR-34c in prostate cancer [10], miR-34a and -34c in colon [11] and lung cancer [12], miR-34a in neuroblastoma [13], and miR-34a and -34b in breast cancer [14, 15]. Many studies report tumor suppressor-like effects of miR-34, for instance in ovarian cancer [16], prostate cancer [10], and neuroblastoma cells [5], putatively by regulating the expression of common miR-34 targets such as *CCND1* [17], *CCNE2* [18], *CDK4* [18, 19], *CDK6* [17, 20], *MET* [18, 19, 21, 22] and *E2F3* [5, 20].

A recent study with prostate cancer PC3 cells revealed that miR-34c expression also resulted in downregulation of protein kinase Cα (PKCα) mRNA [21]. In addition, five target prediction tools (MiRanda [23], DIANAmT [24], miRWALK [25], PICTAR5 [26] and Targetscan [27] predict *PRKCA* as a putative miR-34c target. From a breast cancer perspective this could be of relevance since PKCα expression has been reported to be important for optimal breast cancer cell proliferation [28, 29], support a cancer stem cell-like breast cancer cell population [30] and to predict poorer survival [28].

Taken together, these facts led us to investigate putative suppressive effects of miR-34c on growth properties of breast cancer cells. We found that miR-34c overexpression both blocks the proliferation of cultured basal-like breast cancer cells and induces cell death, although this was not mediated by PKCα downregulation.

## Methods

### Cell Culture

All cell lines were obtained from American Type Culture Collection. MDA-MB-231, MDA-MB-468, BT-549 and T47D breast cancer cells were maintained in RPMI 1640 medium (HyClone, Thermo Scientific) supplemented with 10% fetal bovine serum (Saveen & Werner AB), 1 mM sodium pyruvate (HyClone, Thermo Scientific) and 100 IU/ml penicillin-streptomycin solution (HyClone, Thermo Scientific). The media for BT-549 cells were additionally supplemented with 0.01 mg/ml insulin (Novo Nordisk A/S) and for T47D with 1% glucose.

### Transfections

For miRNA transfections, cells were seeded at 50–60% confluency and grown in complete medium without antibiotics for 24 h. Cells were thereafter transfected for 5 hours with miRIDIAN microRNA Mimic (80 nM probe, Dharmacon, Lafayette, CO, USA) using 2 μl/ml Lipofectamine 2000 (Invitrogen) in Opti-MEM I (Invitrogen) followed by 96 hour incubation in complete medium, roughly according to the manufacturer’s recommendations. Control experiments were performed in parallel, transfecting cells with miRIDIAN microRNA Mimic Negative Control (Dharmacon). Transfection with 40 nM siRNA (Stealth RNAi, Invitrogen) was performed for 72 hours (sequences are listed in Table 1) according to the manufacturer’s protocol.

**Table 1:** **siRNA nucleotides**

siRNA oligonucleotide	Sequence
Control 48% GC	UUACGGAUCGACUUAAGCCGUUGCA
CDC23 I	GCUGCCCAGUGUUACAUCAAAUAUA
CDC23 II	UAUAUUUGAUGUAACACUGGGCAGC
CDC23 III	CCAAGCUCGAGAACUUGAUGGAUUU

### Thymidine incorporation

Cells were seeded in triplicates at a density of 5 × 10^4^ cells per well in 12-well plates and transiently transfected for 5 hours. Cells were incubated with 1 μCi/ml [^3^H]-thymidine for 6 hours before harvesting the cells with 10 mM EDTA. The amount of radioactivity was measured with a Tri-carb 2810TR liquid scintillation analyzer (Perkin Elmer).

### Cell cycle analysis

MDA-MB-231, MDA-MB-468 and BT-549 cells were seeded at a density of 150,000 cells per 35-mm cell culture dish and transiently transfected for 5 hours. Subsequently, cells were trypsinized and fixed in 70% ethanol for 20 minutes at −20°C, washed in PBS, and incubated with a solution containing 3.5 μM Tris- HCl pH 7.6, 10 mM NaCl, 50 μg/ml propidium iodide (PI), 20 μg/ml RNase, and 0.1% igepal CA-630 for 20 minutes on ice to label DNA. 10,000 events were acquired on the FL-2 channel for the PI signal. Sample acquisition and analyses were performed with CellQuest or FACSuite software (BD Biosciences).

### Annexin V analysis

MDA-MB-231 and BT-549 cells were seeded at a density of 150,000 cells per 35-mm cell culture dish, and MDA-MB-468 cells were seeded at 200,000 cells per 35-mm cell culture dish and transfected for 5 hours. After 96 hour incubation in complete medium, floating cells, pooled with trypsinized adherent cells, were stained with Annexin V-allophycocyanin (APC; BD Pharmingen) according to the supplier’s protocol, and the amount of bound Annexin V-APC was quantified with a FACSCalibur cytometer (BD Biosciences). 10,000 events were acquired on the FL-4 channel for the Annexin V-APC signal.

### Real-time qPCR

Total RNA was extracted from MDA-MB-231, MDA-MB-468 and BT549 cells with the RNeasy kit (Qiagen), and potential DNA contamination was eliminated with the RQ1 RNAse-Free DNase (Promega). Two micrograms of total RNA was used for cDNA synthesis with MultiScribe Reverse Transcriptase (Applied Biosystems). The cDNA was thereafter amplified by qPCR for evaluation of relative mRNA expression levels in an Applied Biosystems 7300 real-time quantitative PCR system using the SYBR Green PCR Master Mix (Applied Biosystems). The mRNA expression data were normalized to three reference genes (*SDHA*, *UBC* and *YWHAZ*). For relative quantification of gene expression, the comparative Ct method was applied. The sequences of primers are listed in Table 2.

**Table 2:** **qPCR primers**

Primers for qPCR	Sequence 5’ to 3a
**SDHA** forward	TGGGAACAAGAGGGCATCTG
**SDHA** reverse	CCACCACTGCATCAAATTCATG
**YWHAZ** forward	ACTTTTGGTACATTGTGGCTTCAA
**YWHAZ** reverse	CCGCCAGGACAAACCAGTAT
**UBC** forward	ATTTGGGTCGCGGTTCTTG
**UBC** reverse	TGCCTTGACATTCTCGATGGT
**PRKCA** forward	AAACATCTCCACCCAAGACG
**PRKCA** reverse	AATCCCTCCCTGCTCACTCT
**CCND1** forward	CCCTCGGTGTCCTACTTCAA
**CCND1** reverse	CTCCTCGCACTTCTGTTCCT
**CDCK4** forward	TGTGGAGTGTTGGCTGTATCTT
**CDCK4** reverse	GGTCGGCTTCAGAGTTTCC
**CDCK6** forward	TGGTGCCTCCTCTTGTCTG
**CDCK6** reverse	CTGCCTGTTCCCACTACTCC
**CDC23** forward	CGGAGTTGGCTTTCTCTCTC
**CDC23** reverse	CCTGGGCATCTTCCTCTGTA

For analysis of miR-34b/c expression levels, total RNA was extracted from MDA-MB-231, MDA-MB-468 and T47D cells with Trizol according to manufacturer’s instructions (Invitrogen). Small RNAs were reversely transcribed with miRNA specific primers, quantified by the TaqMan MicroRNA assays (Applied Biosystems) and normalized to two reference genes (*RNU44* and *U47*).

### Western blot analysis

Cells were lysed in radioimmune precipitation assay buffer (10 Mm Tris–HCl (pH 7.2), 160 mM NaCl, 1% Triton X-100, 1% sodium deoxycholate, 0.1% SDS, 1 mM EDTA, and 1 mM EGTA) containing 40 μl/ml Complete protease inhibitor (Roche Applied Science) and incubated on ice for 30 min. Lysates were cleared by centrifugation at 14,000 × g for 10 min at 4°C, diluted in sample buffer containing β-mercaptoethanol, and boiled for 5 min. Protein concentration was determined by Bradford assay, equal amount of proteins were electrophoretically separated on either 10% or 12% NuPAGE Novex BisTris gels (Invitrogen) and transferred to polyvinylidene difluoride membranes (Millipore). Membranes were blocked with phosphate-buffered saline containing 5% nonfat milk and probed with antibodies to Cyclin D1 and PKCα (1:500; Santa Cruz Biotechnology), CDK4 (1:1000; Millipore), CDK6 (1:1000; Cell Signaling Technology), CDC23 (1:1000, Abcam) and actin (1:1000; MP Biomedicals). Proteins were visualized with horseradish peroxidase-labeled secondary antibody (Amersham Biosciences) using the SuperSignal system (Pierce) as substrate. Chemiluminescence was detected using a CCD camera (Fujifilm).

### Data analysis

HiSeq miRNA expression data of 658 breast tumors and 86 normal breast tissue samples and mRNA data from corresponding samples were downloaded from the TCGA database (http://cancergenome.nih.gov/). The data used were downloaded in December 2013. The tumors were clustered based on mRNA expression data using the hclust function in R. Survival analyses were performed on the 310 breast tumors that had follow up data using the Survival package. The TCGA “New tumor event” variable (recurrence) defined as new tumor event after initial treatment was used as end point for survival analyses. Pairwise comparisons were evaluated with a t-test.

## Results

### Expression of miR-34 in breast cancer

**Figure 1:**
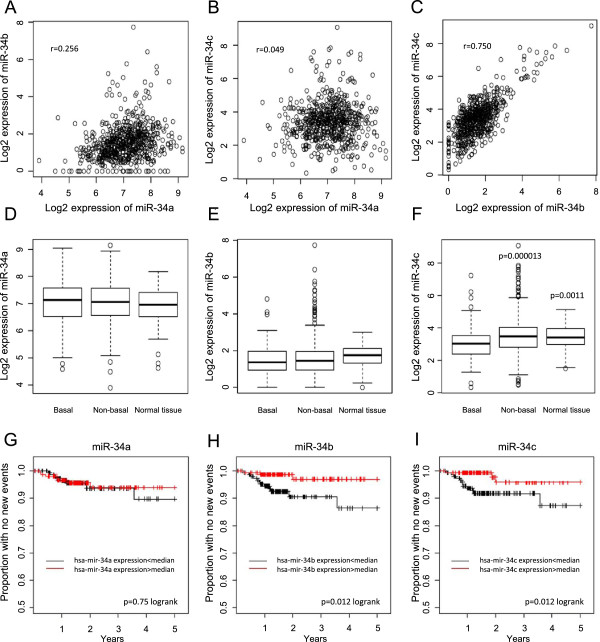
**Analysis of miR-34 family members using breast cancer TCGA data.** Pair-wise scatter-plots of the expression of miR-34a, −34b and -34c in 658 miRNA HiSeq breast tumor samples from TCGA **(A-C)**. Box plots demonstrate log2 expression levels of miR-34a **(D)**, miR-34b **(E)** and miR-34c **(F)** in basal-like tumors, non-basal-like tumors and non-malignant breast tissue. Indicated p-values were calculated with a t-test comparing the group with the basal-like tumor samples. Kaplan-Meier analysis curves were constructed using the 310 TCGA tumors that had miRNA HiSeq data and follow-up data **(G-I)**. The expression data were divided based on median expression and new tumor event was used as end point.

To assess putative roles of miR-34 family members in breast cancer, miRNA HiSeq expression data from 658 tumors and 86 normal breast tissue samples from the TCGA (The Cancer Genome Atlas) database were used. There was a clear correlation between miR-34b and miR-34c levels, whereas neither of them displayed a strong correlation with miR-34a (Figure 1A-C). This likely reflects the fact that miR-34b and 34c are located at the same locus on chromosome 11q23 whereas miR-34a is located at 1p36. The TCGA tumors were clustered using mRNA data, which separated the tumors in two major groups which correspond to basal-like and non-basal-like tumors. For miR-34c, lower levels were seen in basal-like tumors compared both to non-basal-like tumors and to normal breast tissue, whereas no substantial difference was observed for miR-34a or miR-34b (Figure 1D-F).The miR-34 expression data from the 310 tumors with follow-up information were split into two groups based on the median of the expression and survival curves with new tumor event as end point were generated (Figure 1G-I). No obvious difference between tumors expressing high or low levels of miR-34a could be seen. However, for both miR-34b and miR-34c, there was a difference in new tumor events between the low- and high-expressing groups, with more new tumor events in the group with miR-34 expression below median.

The levels of miR-34b and miR-34c were analyzed in two ER-negative (MDA-MB-231 and MDA-MB-468) and one ER-positive (T47D) cell line. In the ER-negative MDA-MB-468 cell line the levels were barely detected whereas the magnitude of expression was similar in the other cell lines (Additional file 1).

### Expression of miR-34c decreases cell proliferation in breast cancer cells

**Figure 2:**
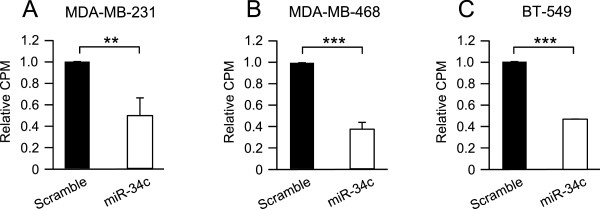
**Effect of miR-34c on proliferation of breast cancer cells.** MDA-MB-231 **(A)**, MDA-MB-468 **(B)** and BT-549 **(C)** breast cancer cells were transiently transfected with either a miR-34c mimic or negative control for 96 h and then subjected to thymidine incubation for 6 hours. Data (mean ± SEM from three separate experiments) are expressed as [^3^H]-thymidine incorporation related to control cells. Asterisks indicate statistically significant differences (* p < 0.05, ** p < 0.01, *** p < 0.001, Student’s *t*-test) compared to control cells.

Only miR-34c was expressed at lower levels in basal-like tumors. To investigate putative effects of miR-34c on breast cancer cell growth, three basal-like breast cancer cell lines, MDA-MB-231, MDA-MB-468 and BT-549, were transfected with miR-34c mimic or a scramble control oligonucleotide followed by [^3^H]-thymidine incorporation. In concordance with effects in other cell types [10, 16, 18], a suppressed proliferation with around 50% was observed following miR-34c transfection in all evaluated cell lines (Figure 2A-C).

### Effect of miR-34c on cell cycle distribution

**Figure 3:**
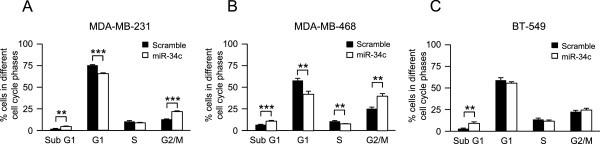
**Effect of miR-34c on cell cycle distribution of breast cancer cells.** Following transfection of MDA-MB-231 **(A)**, MDA-MB-468 **(B)** and BT-549 **(C)** breast cancer cells with miR-34c mimic or negative control for 96 h, nuclei were stained with propidium iodide solution and analyzed for DNA content by flow cytometry. Data (mean ± SEM, n = 5) represent percentage cells in different phases of the cell cycle with miR-34c related to scramble treatment. Asterisks indicate statistically significant differences (* p < 0.05, ** p < 0.01, *** p < 0.001, Student’s *t*-test) compared to control cells.

The suppressed [^3^H]-thymidine incorporation suggests that miR-34c may influence the cell cycle. The cell cycle distribution was thus analyzed by FACS following nuclear staining with propidium iodide (Additional file 2). Ectopic expression of miR-34c induced an accumulation of cells in the G2/M phase compared to control in MDA-MB-231 (Figure 3A) and MDA-MB-468 (Figure 3B) cells. A similar tendency was observed for BT-549 cells (Figure 3C). In all three cell lines a significant increase in sub-G1 phase was detected along with a reduction of cells in G1 phase (Figure 3A-C), but the G1-arrest that has been reported for other cell types [18, 31] was not detected in the breast cancer cells.

### Expression of miR-34c leads to increased cell death in breast cancer cells

**Figure 4:**
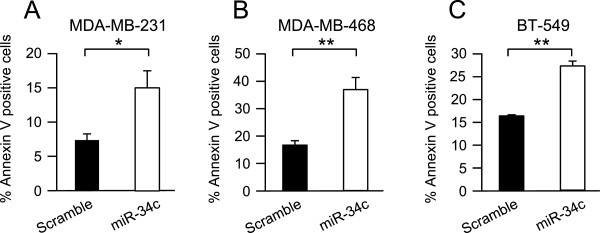
**Effect of miR-34c on apoptosis of breast cancer cells.** MDA-MB-231 **(A)**, MDA-MB-468 **(B)** and BT-549 **(C)** breast cancer cells were transiently transfected with either miR-34c mimic or negative control for 96 h. After 96 h, cells were subjected to Annexin V-APC staining and flow cytometry analysis. Data (mean ± SEM, n = 2-3) represent percent AnnexinV-positive cells with miR-34c related to scramble treatment. Asterisks indicate statistically significant differences (* p < 0.05, Student’s *t*-test) compared to control cells.

The increase in nuclei in sub-G1 phase induced by miR-34c suggests that high expression of miR-34c can lead to induction of cell death. This was therefore analyzed with an Annexin V assay. In all cell lines, the number of Annexin V-positive cells was roughly doubled, indicating an increased cell death, following miR-34c overexpression (Figure 4 A-C).

### Evaluation of miR-34c targets in breast cancer

**Figure 5:**
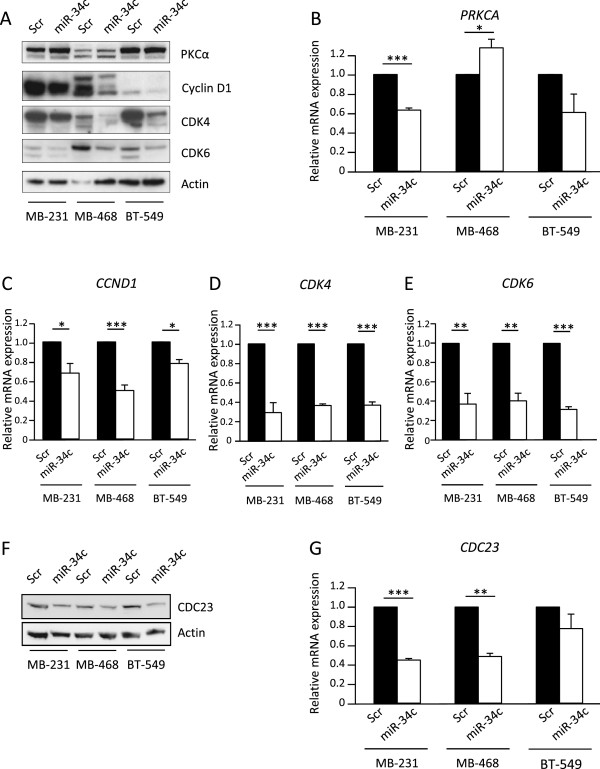
**Evaluation of miR-34c targets.** Following transfection of MDA-MB-231, MDA-MB-468 and BT-549 (breast cancer cells with miR-34c mimic or negative control for 96 h, cells were analyzed for expression of *PRKCA* **(B)**, *CCND1* **(C)**, *CDK4* **(D)**, *CDK6* **(E)** or *CDC23* **(G)** mRNA with real-time quantitative PCR or for protein expression with Western blot **(A** **and** **F)**. Data represent mean ± SEM from 2–3 independent experiments and the blots shown are representative of three independent experiments. Asterisks indicate statistically significant differences (* p < 0.05, ** p < 0.01, *** p < 0.001, Student’s *t*-test) compared to control cells.

As mentioned in the introduction, *PRKCA* is a predicted miR-34c target. Since PKCα is important for optimal breast cancer cell proliferation [28–30] we analyzed the effects of miR-34c on PKCα expression. We could not detect any effects on the protein levels (Figure 5A) despite the observation that *PRKCA* mRNA levels in MDA-MB-231 and MDA-MB-468 were affected by miR-34c (Figure 5B). This suggests that PKCα downregulation is not a mediator of the effects seen by miR-34c in breast cancer cells.

To obtain some insight into putative mediators of the miR-34c effect, we next analyzed mRNA and protein levels of the cell cycle regulators cyclin D1, CDK4 and CDK6, which have been identified as targets of miR-34c and its relatives [17, 18, 32]. In line with this, we found that miR-34c overexpression resulted in decreased protein levels of cyclin D1, CDK4 and CDK6 in all cell lines (Figure 5A). A significant decrease in their mRNA levels was also detected (Figure 5C-E).

Cyclin D1, CDK4, and CDK6 are mainly considered to be important in the G1/S transition but the main effect observed following miR-34c treatment was actually an arrest in G2/M. We thus analyzed the protein and mRNA levels of CDC23 which is an important regulator of mitotic progression. *CDC23* mRNA has been shown to be pulled-down as well as downregulated by miR-34a in colorectal cancer cells [33] and downregulated by miR-34c in prostate cancer cells [21]. In addition, *CDC23* is predicted to contain a putative miR-34c binding site in the 3’UTR by five target prediction tools (MiRanda [23], DIANAmT [24], miRWALK [25], PICTAR5 [26] and Targetscan [27], indicating that *CDC23* might be a direct target of miR-34c. A decrease both in protein and mRNA levels of CDC23 was indeed observed in all cell lines following miR-34c expression (Figure 5F-G) suggesting that suppression of CDC23 may mediate some miR-34c effects, either as a direct target of miR-34c or via an indirect mechanism.

**Figure 6:**
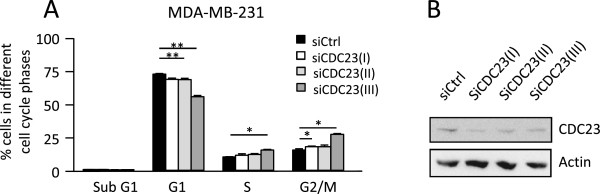
**Effects of** ***CDC23*** **down regulation on cell cycle distribution.** MDA-MB-231 cells were treated for 72 h with three separate siRNAs targeting *CDC23*. Thereafter the cell cycle distribution was analyzed with flow cytometry **(A)** and CDC23 protein levels were analyzed with Western blot **(B)**. The data in A are mean ± SEM from three separate experiments. Asterisks indicate statistically significant differences (* p < 0.05, *** p < 0.001, paired *t*-test) compared to control cells.

To analyse whether suppression of CDC23 levels is sufficient to elicit some miR-34c effects, MDA-MB-231 cells were treated with siRNA targeting *CDC23* mRNA (Figure 6). This resulted in fewer cells in the G1 and increases in the G2/M phase. However, no effect on cells in the sub-G1 phase could be seen.

## Discussion

In cancers, dysregulation of miRNA is a common feature that can affect downstream targets and further influence tumorigenic events such as proliferation, metastasis and apoptosis [34]. Family members of miR-34 have been reported to be downregulated in several different cancers, including prostate [10], neuroblastoma [13], colon [11], lung [12] and breast [14, 15]. In addition, epigenetic silencing through CpG methylation [35, 36] and homozygous deletions affecting the miR-34a and miR-34b/c loci (1p36 and 11q23, respectively) has been identified in neuroblastoma and other tumors [5, 7, 37–39].

Our analyses of TCGA data indicate that low levels of miR-34b and/or miR-34c may predict a worse outcome of breast cancer. However, the data are not in line with previous reports indicating that miR-34a and miR-34b are downregulated in breast cancer [40–42]. It was only for miR-34c in basal-like breast cancers that lower expression levels could be seen. This indicates that miR-34c may be the most relevant miR-34 family member to overexpress in basal-like breast cancer cells.

In this study, we have identified an anti-proliferative and pro-apoptotic effect by miR-34c in basal-like breast cancer cells, in concordance with reports from studies in other cancers [16, 21]. Previous studies have pointed out a role for miR-34a [13, 18, 35, 43–45], and in some cases for miR-34c [18, 31], in suppression of the cell cycle, mainly by induction of G1 cell cycle arrest. Our data rather indicate that miR-34c induced a G2/M arrest in breast cancer cells. This is more in line with the miR-34a-promoted mitotic catastrophe and G2/M arrest in irradiated glioblastoma cells [46]. One member of the anaphase-promoting complex (APC), *CDC23*, has been reported to be a target of miR-34a [33] and show a decreased mRNA expression in response to miR-34c in prostate cancer cells [21]. In our analysis we detect a significant decrease of CDC23 both at mRNA and protein levels in response to miR-34c expression. *CDC23* may be a mediator of miR-34c effects, but more specific experiments are needed to settle *CDC23* as a direct miR-34c target. The decrease in G1 and increase in G2/M could be replicated by down regulation of CDC23 supporting the hypothesis that downregulation of CDC23 may mediate some of the observed miR-34c effects. However, there was no effect on cells in the sub-G1 phase suggesting that miR-34c-induced cell death may be mediated by other mechanisms.

PKCα protein levels were not influenced by miR-34c and a downregulation of PKCα is therefore conceivably not involved in the observed effects. However, the *PRKCA* mRNA levels were affected, albeit in different directions depending on cell line. The diverging effects on *PRKCA* mRNA levels suggest that it is less likely a direct target of miR-34c.

We also observed that miR-34c induces death in breast cancer cells. This could be a consequence of a G2/M arrest or involve other mechanisms, such as suppression of the pro-survival factors BCL2 [13, 32] or SIRT1 [47]. The fact that siCDC23 induces a G2/M arrest, but no increasing in sub-G1 phase, indicates that the effects may be separate. Induction of cell death actually seems to be a more general miR-34 effect since they have been shown to lead to increased cell death in several cell types [5, 10, 48]. Along with the growth-suppressing and cell death-inducing effects shown in this study, miR-34c has been shown to reduce the migratory and self-renewing capacity of breast tumor-initiating cells [49] and to inhibit metastatic invasion in vivo [15]. Our study further indicates that miR-34c has tumor-suppressive effects in breast cancer and, together with other reports, this implies miR-34c to be a potential mediator for novel miRNA replacement therapies [50].

## Conclusions

In conclusion, we have detected a suppressive role for miR-34c in breast cancer cell growth and a G2/M cell cycle arrest in response to miR-34c induction. We also identified *CDC23* as a miR-34c-regulated target that could be responsible for the miR-34c-induced cell cycle arrest.

## Electronic supplementary material

Additional file 1: **Expression levels of miR-34b and miR-34c in breast cancer cell lines.** MDA-MB-231, MDA-MB-468 and T47D cells were analyzed for basal expression levels of miR-34b (A) and miR-34c (B). The data are mean ± SEM from three separate experiments. (PDF 25 KB)

Additional file 2: **Effect of miR-34c on cell cycle distribution.** Representative cell cycle profiles of breast cancer cell lines transfected with miR-34c mimic or negative control. Quantifications are given in Figure 3. (PDF 93 KB)

## Abbreviations

miR: microRNA
TCGA: The Cancer Genome Atlas
3’UTR: 3’untranslated region
CCND1: Cyclin D1
CDK4: Cyclin-dependent kinase 4
CDK6: Cyclin-dependent kinase 6
CDC23: Cell division cycle 23
PRKCA: Protein kinase Cα.
