# Supplementary figures and images for: Expression of miR-34c induces G2/M cell cycle arrest in breast cancer cells
Source: BMC Cancer. 2014 Jul 26;14:538. doi: 10.1186/1471-2407-14-538 (PMC4125691; doi:10.1186/1471-2407-14-538)

**A**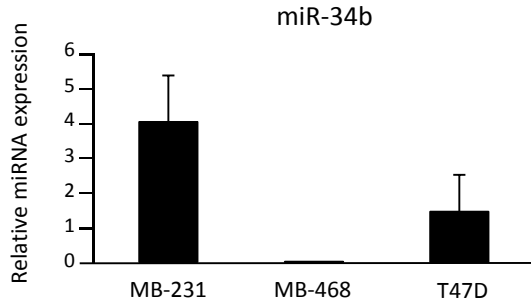**B**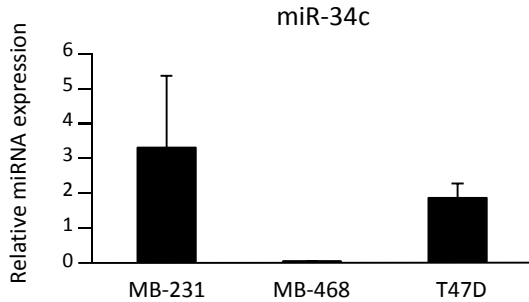

Supplement: Supplementary file 1 — Additional file 1: Expression levels of miR-34b and miR-34c in breast cancer cell lines. MDA-MB-231, MDA-MB-468 and T47D cells were analyzed for basal expression levels of miR-34b (A) and miR-34c (B). The data are mean ± SEM from three separate experiments. (PDF 25 KB) [file 12885_2014_4733_MOESM1_ESM.pdf]

A

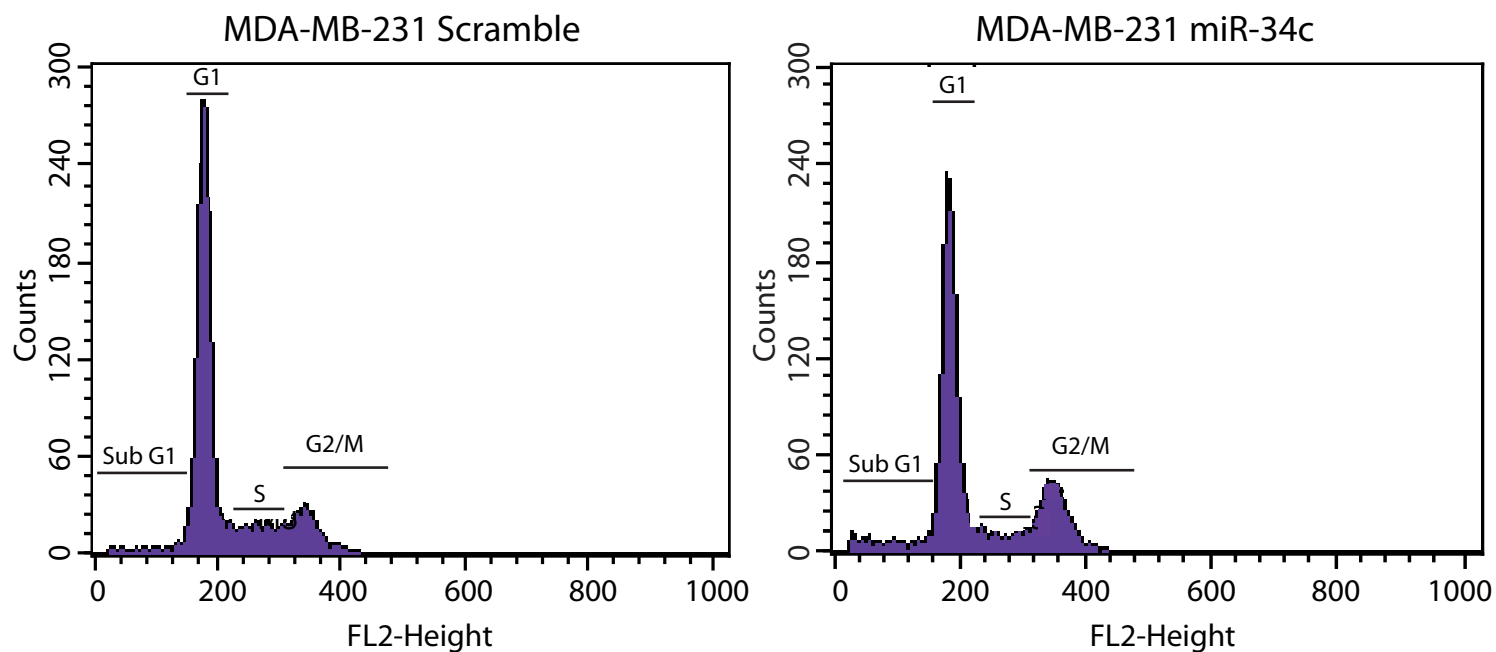

B

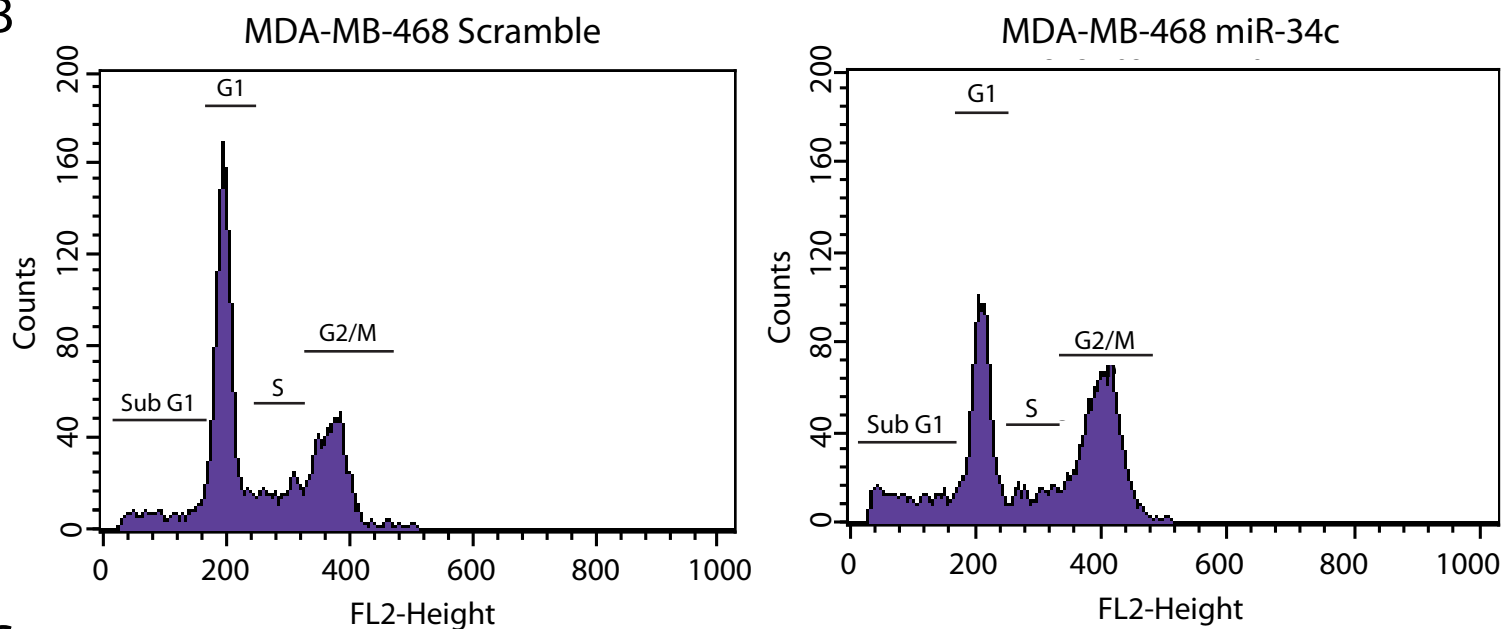

C

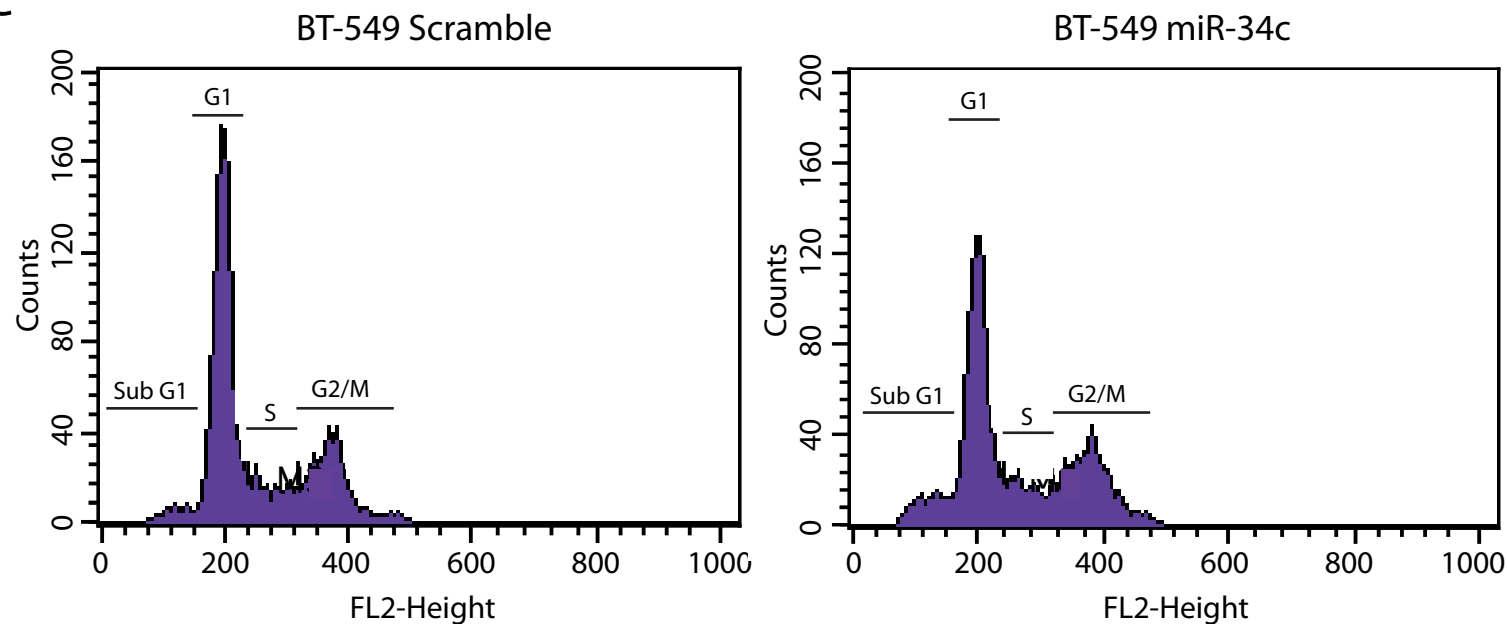

Supplement: Supplementary file 2 — Additional file 2: Effect of miR-34c on cell cycle distribution. Representative cell cycle profiles of breast cancer cell lines transfected with miR-34c mimic or negative control. Quantifications are given in Figure 3. (PDF 93 KB) [file 12885_2014_4733_MOESM2_ESM.pdf]
